# Preclinical radiation dosimetry for the novel SV2A radiotracer [^18^F]UCB-H

**DOI:** 10.1186/2191-219X-3-35

**Published:** 2013-05-07

**Authors:** Florian Bretin, Geoffrey Warnock, Mohamed Ali Bahri, Joël Aerts, Nathalie Mestdagh, Tim Buchanan, Anne Valade, Frédéric Mievis, Fabrice Giacomelli, Christian Lemaire, André Luxen, Eric Salmon, Alain Seret, Alain Plenevaux

**Affiliations:** 1 Cyclotron Research Centre, University of Liège, Allée du 6 Août, Building B30, Sart Tilman, Liège 4000, Belgium; 2 UCB Pharma, NewMedicines-Neurosciences Discovery Research, Braine-l’Alleud 1420, Belgium; 3 UCB Pharma, NewMedicines-Neurosciences Discovery Medicine, Braine-l’Alleud 1420, Belgium

**Keywords:** Dosimetry, Preclinical microPET, Organ harvesting, SV2

## Abstract

**Background:**

[^18^F]UCB-H was developed as a novel radiotracer with a high affinity for synaptic vesicle protein 2A, the binding site for the antiepileptic levetiracetam. The objectives of this study were to evaluate the radiation dosimetry of [^18^F]UCB-H in a preclinical trial and to determine the maximum injectable dose according to guidelines for human biomedical research. The radiation dosimetry was derived by organ harvesting and dynamic micro positron emission tomography (PET) imaging in mice, and the results of both methods were compared.

**Methods:**

Twenty-four male C57BL-6 mice were injected with 6.96 ± 0.81 MBq of [^18^F]UCB-H, and the biodistribution was determined by organ harvesting at 2, 5, 10, 30, 60, and 120 min (*n* = 4 for each time point). Dynamic microPET imaging was performed on five male C57BL-6 mice after the injection of 9.19 ± 3.40 MBq of [^18^F]UCB-H. A theoretical dynamic bladder model was applied to simulate urinary excretion. Human radiation dose estimates were derived from animal data using the International Commission on Radiological Protection 103 tissue weighting factors.

**Results:**

Based on organ harvesting, the urinary bladder wall, liver and brain received the highest radiation dose with a resulting effective dose of 1.88E-02 mSv/MBq. Based on dynamic imaging an effective dose of 1.86E-02 mSv/MBq was calculated, with the urinary bladder wall and liver (brain was not in the imaging field of view) receiving the highest radiation.

**Conclusions:**

This first preclinical dosimetry study of [^18^F]UCB-H showed that the tracer meets the standard criteria for radiation exposure in clinical studies. The dose-limiting organ based on US Food and Drug Administration (FDA) and European guidelines was the urinary bladder wall for FDA and the effective dose for Europe with a maximum injectable single dose of approximately 325 MBq was calculated. Although microPET imaging showed significant deviations from organ harvesting, the Pearson’s correlation coefficient between radiation dosimetry derived by either method was 0.9666.

## Background

Epilepsy is a chronic neurological disorder characterized by seizures and abnormal electroencephalographic activity. It affects people of all ages with more than 50 million cases worldwide. In contrast to other antiepileptic drugs, levetiracetam (Keppra®, UCB Pharma Ltd., Slough, Berkshire, UK) has a unique mechanism of action, binding to the neuronal synaptic vesicle protein 2A (SV2A) in the brain [1]. SV2 proteins are critical to proper nervous system function, and they have been demonstrated to be involved in vesicle trafficking. However, the specific role of SV2A in epilepsy remains unclear. The newly developed fluorine-18 radiolabelled positron emission tomography (PET) imaging agent [^18^F]UCB-H shows a nanomolar affinity for the human SV2A protein and will be of great value in studying the function of SV2A in diseases of the central nervous system [2].

A prerequisite to the use of a novel radiotracer in human clinical trials and good clinical practice is a preclinical dosimetry study in animals. This enables the prediction of dose limits in humans that will keep radiation doses below harmful limits while still producing diagnostically beneficial images. Radiation estimates for humans derived from small animals are traditionally obtained by *ex vivo* tissue distribution (TD) studies, where organs are harvested post-injection at several time points to establish the biodistribution. However, dynamic imaging approaches in small animals using microPET are a promising alternative, because the complete biodistribution of the radiopharmaceutical can be obtained within a single *in vivo* scan with a much higher time resolution. The aims of this study were to predict the radiation dose given to humans based on the distribution of [^18^F]UCB-H in mice and to determine the maximum injectable dose according to radiation guidelines in biomedical research. Additionally, the TD and microPET approaches to assess the biodistribution were investigated and compared.

## Methods

### Chemistry

[^18^F]UCB-H was obtained in a four-step radiosynthesis. Briefly, this consisted of nucleophilic labeling of a pyridine precursor, reductive amination of the labeled product, and internal cyclisation. Specific activity was higher than 500 MBq/μg at the end of synthesis.

### Animals

Male C57BL-6 mice were initially obtained at 5 weeks of age from Charles River Laboratories (Brussels, Belgium) and subsequently bred at the Animal Facility of the GIGA-University of Liège (BE-LA 2610359). The animals were housed under standard 12h:12h light/dark conditions with food and water available *ad libitum*. All experimental procedures and protocols used in this investigation were reviewed and approved by the Institutional Animal Care and Use Committee of the University of Liège.

### Tissue distribution

The data acquisition and analysis were conducted in accordance with MIRD pamphlet no. 16 [3]. For the TD of [^18^F]UCB-H, 24 male C57BL-6 mice (23.96 ± 1.31 g [20.6 to 26.1 g]) were injected with an iv bolus of 6.96 ± 0.81 MBq [5.18 to 8.06 MBq] [^18^F]UCB-H via the tail vein under isoflurane anesthesia. Anesthesia was maintained until sacrificed by decapitation after 2, 5, 10, 30, 60, and 120 min (*n* = 4 for each time point). Blood samples were obtained (0.5 to 1 ml), and the following organs were harvested by dissection: the brain, bone (femur), liver (partial), kidney, heart, spleen, intestine (partial), pancreas, adrenals, testes, skin (partial), muscle (partial/thigh), stomach, lung, and bladder. All organs were weighed (NewClassic ML, Mettler Toledo, Switzerland), and the activity was quantified using a gamma well counter (Cobra II Auto-Gamma, Perkin-Elmer, Waltham, MA, USA), resulting in the average activity per gram of tissue in mice. The gamma well counter was calibrated measuring a known quantity of ^18^F activity (quantified using a calibrated gamma spectrometer: high purity 30% germanium GR3020 from Canberra Industries, Meriden, CT, USA), thereby obtaining a calibration factor between real and measured activity. The measurement uncertainty was evaluated to be ±5%.

### Dynamic microPET imaging

Dynamic microPET imaging was performed on five male C57BL-6 mice with an average weight of 29.72 ± 6.70 g [24.2 to 40.8 g]. 9.19 ± 3.40 MBq [3.84 to 14.20 MBq] [^18^F]UCB-H was administered via the tail vein as bolus. All procedures were performed under isoflurane anesthesia. Co-registered micro-computed tomography (CT) images were acquired with an eXplore 120 microCT (Gamma Medica, USA/GE Healthcare, Sevenoaks, Kent, UK) [4] to obtain anatomical information for segmentation. An iodine-based contrast agent (1:10 dilution of iobitridol-XENETIX300, Guerbet, Roissy, France) was administered in the peritoneal cavity prior to microCT to aid full organ segmentation. All microCT images were reconstructed using Feldkamp’s filtered backprojection algorithm [5] with a cutoff at the Nyquist frequency and an isotropic voxel size of 100 μm. The same MINERVE animal cell bed (Equipement Veterinaire Minerve, Esternay, France) was used in both imaging modalities to provide anesthesia, physiological control, monitoring of respiration, and simplified co-registration of the images. Dynamic microPET scans over 120 min were acquired using a Siemens Concorde Focus 120 microPET (Siemens, Munich, Germany) [6] and followed by transmission measurement with ^57^Co point source. In order to obtain dynamic imaging data for as many organs as possible, the field of view of the scanner was positioned on the chest and lower body disregarding the brain. The list-mode data were histogrammed into three-dimensional (3D) sinograms by Fourier rebinning [7] and reconstructed by filtered backprojection with a ramp filter cutoff at the Nyquist frequency. Corrections for randoms, dead time, and attenuation were applied but not scatter correction, since it was shown by Bahri et al. [8] that no benefit for quantification is gained by applying scatter correction for the Focus-F120. No partial volume correction was performed on the acquired data. A set of 3D images was reconstructed in a 256 × 256 × 95 matrix with a pixel size of 0.4 × 0.4 × 0.8 mm. The dynamic time framing was as follows: 6 × 5 s, 6 × 10 s, 3 × 20 s, 5 × 30 s, 5 × 60 s, 8 × 150 s, 6 × 300 s, and 6 × 600 s, and all data were decay corrected to the beginning of each individual frame.

MicroCT and microPET images were co-registered using a landmark-based approach. Volumes of interest (VOIs) were drawn manually by a single observer on the microCT images using PMOD (Version 3.306, PMOD Technologies, Zurich, Switzerland). This allowed the delineation of the following organs: the heart, lung, liver, kidneys, and testes (referred to as ‘dynamic whole’). The average organ activity per volume was obtained from the co-registered microPET images. Additionally, VOIs in the shape of spheres with a radius of 2 mm were placed in the center of the same organs to limit the impact of partial volume effects on the quantification and used for dosimetry (referred to as ‘dynamic single’). For the heart, one VOI was positioned over the myocardium and parts of the chambers; for the liver, two VOIs were placed in the center of the organ; for lungs, kidneys, and testes, one VOI was placed in the center of each organ (i.e., left and right wing for lungs, left and right organ for kidneys and testes). MicroPET calibration for conversion of counts/pixel in Bq/ml was performed as recommended by the manufacturer. Accuracy of the technique has been reported to be better than 2% [8].

### Dosimetry analysis

When using animal data to predict human dosimetry, an interspecies extrapolation is necessary to account for differences between animal and human. A commonly used method in preclinical imaging is that of Kirshner et al. [9], which is based on a linear scaling of the percent uptake in the animal by the ratio of the organ weights and total body weights in both species:

$$\left[{\left(\frac{\%}{g_{\mathrm{organ}}}\right)}_{\mathrm{animal}}•{\left(kg_{\mathrm{TBweight}}\right)}_{\mathrm{animal}}\right]•{\left(\frac{g_{\mathrm{organ}}}{kg_{\mathrm{TBweight}}}\right)}_{\mathrm{human}}={\left(\frac{\%}{\mathrm{organ}}\right)}_{\mathrm{human}}$$

The organ uptake in percentage of injected dose multiplied by the body weight in kilograms is assumed to be constant across species, allowing for the calculation of equivalent organ activities in humans from animal data. Since the organ-to-body weight ratio is necessary to extrapolate to humans and to create consistent time-activity curves from the obtained average organ activity per volume or gram of tissue, a ‘standard mouse’ was created using six C57BL-6 mice. This ‘standard mouse’ allowed both organ density and the ratio of organ weight to body weight to be calculated and a species average assumed. The mice were weighed and then dissected to determine the organ weight and density by microCT for volume quantification. The mean total body weight was 32.28 ± 3.98 g [28.6 to 39.8 g]. The following organs were harvested: the brain, liver, kidney, heart, spleen, testes, and lung. The organ volume was obtained from the microCT images by a threshold-based segmentation approach. All weights were finally scaled to a bodyweight of 35 g. Only data from organs where the ratio of organ weight to bodyweight was known were used for dosimetry.

Average organ activity per mass or volume was normalized for injected dose and multiplied by organ weights or organ volumes, respectively. Activity levels were linearly interpolated, and only physical decay was assumed after the last time point up to 10 h post-injection. Thus, a homogenous activity distribution within each organ was assumed, and biological clearance after 2 h post-injection was neglected, assuming the activity was ‘trapped’ within the source organ. The time-activity curves (TACs) were then extrapolated from animal values to human values using Equation 1. Human organ weights and body weight were taken from the standard 70-kg adult male hermaphroditic phantom implemented in the human dosimetry software OLINDA/EXM (version 1.1) [10] and animal organ weights from the standard mouse as described above. In addition, TACs that were calculated based only on animal organs (i.e., no extrapolation to human values) were used to calculate additional dose estimates to provide an estimation window for the human dosimetry (referred to as ‘NE’ for no extrapolation). Urine excretion data were modeled using the implemented dynamic voiding bladder module [11] in OLINDA/EXM. A voiding interval of 4 h was assumed, and fractions of 0.5 to 0.3 of total injected activity leaving the body via the urinary excretion system with a biological half-life of 3 h were defined and separately implemented. The same scenarios were implemented using a voiding interval of 2 h to provide data for the impact of a possible pre-scan hydration of the subject to decrease the bladder dose. The residence time for each organ, which is equal to the number of disintegrations within the source organ, was calculated by trapezoidal numerical integration of the TACs in MATLAB (version 7.12.0) as proposed in literature [3,12]. Activity from other organs that could not be extrapolated due to the unknown weight-to-body weight ratio was assigned together with all unaccounted for activity to the remainder, which is assumed to be homogenously distributed throughout the remaining body. OLINDA/EXM was used to calculate absorbed doses. The effective doses in the standard 70-kg male human based on the recent tissue weighting factors published in International Commission on Radiological Protection (ICRP) 103 [13] were calculated using Excel (version 2010, Microsoft, Albuquerque, NM, USA) and are presented in the ‘Results’ section. For comparison purposes with other tracers and previous works, the effective doses using old tissue weighting factors from ICRP 60 [14] were also calculated using OLINDA/EXM and were provided as additional files.

Since the TACs of the TD are derived from several time points in different animals, each individual time point has its own standard deviation (SD). Therefore, the SD of residence times derived from the TD was calculated as follows. Two additional animal TACs were constructed using mean ± 1 SD of each individual time point of the activity measurement. These two TACs were then extrapolated to human values as previously described, and the mean residence time corresponding to each TAC was computed [15-17]. This provided a residence time window which is expressed as a coefficient of variation in percent (%CV) from the mean value. The residence time window was used to calculate a minimum and maximum dose for the TD, resulting more in a best and worst case scenario as opposed to a real SD. The mean of best and worst case scenario was used as the error for the measured absorbed dose and reported as %CV of the mean value. For the statistics of the microPET imaging, every scan was treated separately, and mean and standard deviation expressed as %CV were regularly calculated across scans. Correlations between datasets were calculated by the Pearson product–moment correlation coefficient in MATLAB.

## Results

**Figure 1:**
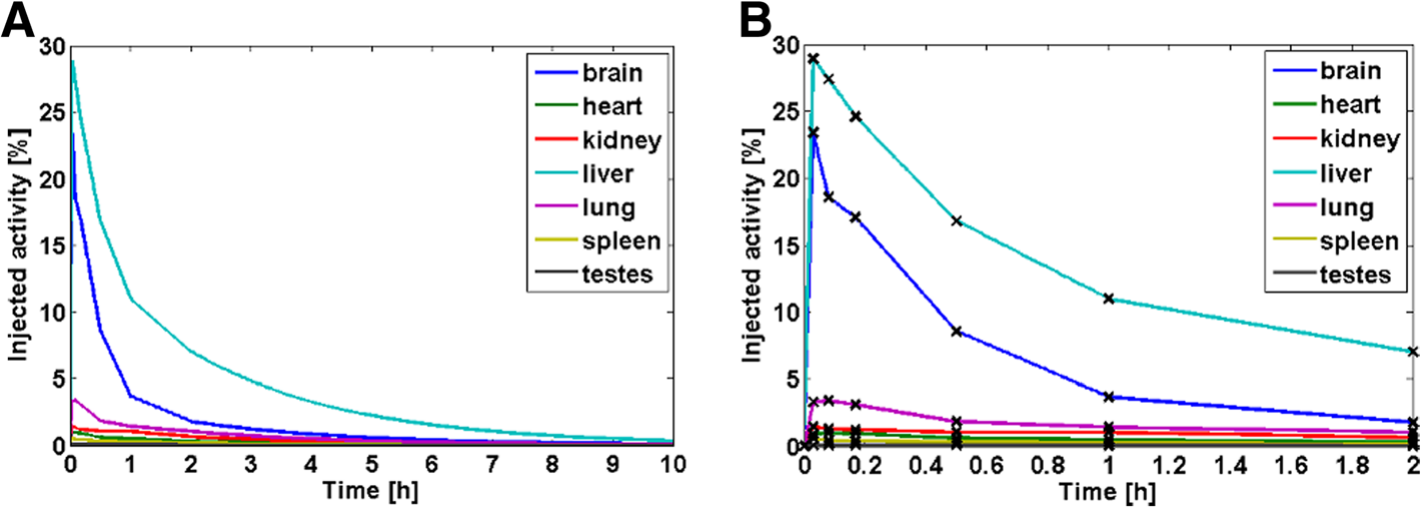
Mean human TACs derived by TD in mice (A: total, B: zoom in).

**Figure 2:**
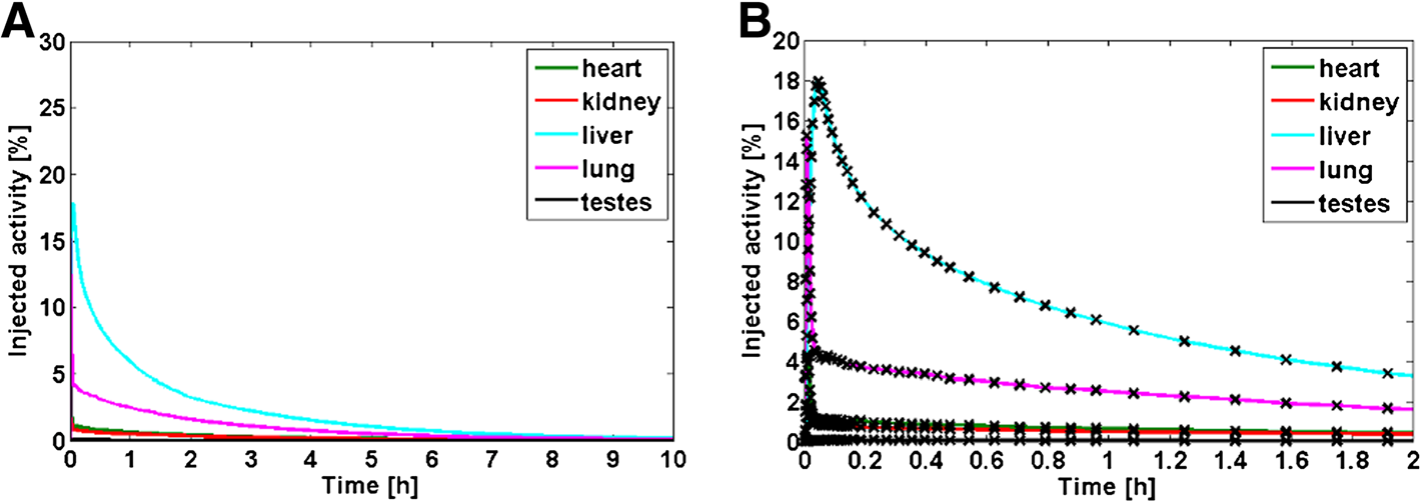
Mean human TACs derived by microPET imaging in mice (dynamic single) (A: total, B: zoom in).

Figures 1 and 2 illustrate the human biodistribution of [^18^F]UCB-H derived from animal TD and from microPET imaging based on sphere segmentation (dynamic single), respectively. In both datasets, the highest initial activity uptake was obtained within the liver with 29% and 18%, respectively. However, brain data could not be derived from PET data, as the brain was outside the scanner’s field of view. Furthermore, the spleen could not be accurately delineated in CT images in the absence of additional contrast agents. TACs from whole organ segmentation (dynamic whole) were provided as Additional file 1 (whole TACs) and Additional file 2 (zoom in).

The mean residence times obtained by the TD and dynamic microPET imaging with both segmentation methods are presented in Table 1. The highest level of disintegrations per organ occurred within the liver with 4.45E-01 h (TD), 1.93E-01 (dynamic whole), and 2.18E-01 h (dynamic single). Bladder values are theoretical values derived using the dynamic bladder module described above.

**Table 1:** Human residence times (h) (bladder fraction 0.5) derived by TD and dynamic imaging based on whole organ segmentation and single spheres

**Organ**	**TD**	**%CV**	**Dynamic whole**	**%CV**	**Dynamic single**	**%CV**
Bladder (theoretical)	3.08E-01	-	3.08E-01	0.0	3.08E-01	0.0
Brain	1.74E-01	18.1	-	-	-	-
Heart wall	1.79E-02	10.1	2.45E-02	11.6	2.39E-02	12.3
Kidneys	3.57E-02	10.5	1.67E-02	9.1	1.90E-02	13.2
Liver	4.45E-01	13.8	1.93E-01	6.7	2.18E-01	6.6
Lung	5.96E-02	9.8	1.13E-01	11.8	9.30E-02	14.8
Spleen	9.04E-03	13.6	-	-	-	-
Testes	2.11E-03	12.7	1.04E-03	12.1	1.12E-03	12.8
Remaining body	1.58E + 00	6.7	1.98E + 00	1.4	1.97E + 00	1.2

**Figure 3:**
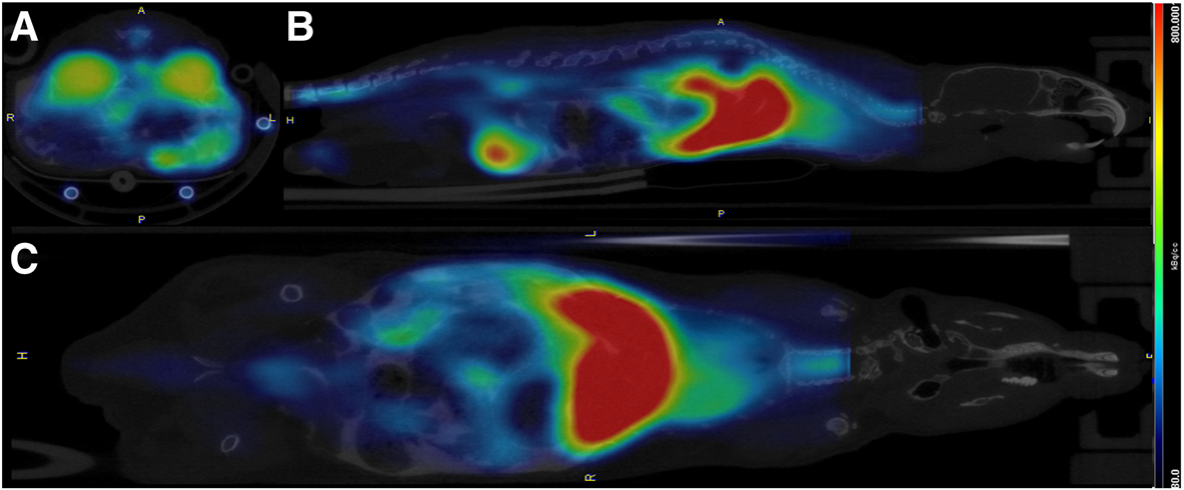
**Transverse (A), sagittal (B), and coronal (C) planes of merged average PET/CT image of a representative subject.** PET image was averaged across all frames and smoothed using a Gaussian kernel of 1 × 1 × 2 mm in PMOD. The color scale was set to the range 80 to 800 kBq/cc in all planes (Brain outside FOV).

A cross section of all anatomical planes of the merged PET/CT image is illustrated in Figure 3. High uptake in liver, kidneys, bladder, and spinal cord can be observed.

The animal-derived dose estimations from the TD method (Table 2) predicted that the highest absorbed dose is received by the urinary bladder wall (modeled theoretical value) with 1.54E-01 ± 5.00E-04 mGy/MBq, followed by the liver with 5.84E-02 ± 7.35E-03 mGy/MBq and the brain with 3.08E-02 ± 5.15E-03 mGy/MBq. The effective dose was 1.88E-02 ± 5.11E-04 mSv/MBq. The highest dose estimate, based on microPET imaging (dynamic single), was received by the urinary bladder wall with 1.56E-01 ± 0.00E-00 mGy/MBq, followed by the liver with 3.14E-02 ± 1.97E-03 mGy/MBq (brain was not imaged). The effective dose was 1.84E-02 ± 3.51E-04 mSv/MBq. Mean dose estimates based on whole organ segmentation are provided as Additional file 3 and effective doses for all three methods obtained using old weighting factors form ICRP 60 as Additional file 4.

**Table 2:** Human absorbed dose (mGy/MBq) and effective dose (mSv/MBq)

**Target Organ**	**TD**	**%CV**	**TD NE**	**Dynamic single**	**%CV**	**Dynamic single NE**
Adrenals	1.43E-02	1.7	1.65E-02	1.36E-02	1.0	1.44E-02
Brain	3.08E-02	16.7	2.05E-02	8.61E-03	1.1	8.27E-03
Breasts	7.71E-03	2.9	7.18E-03	8.88E-03	0.7	8.36E-03
Gallbladder wall	1.86E-02	4.3	2.26E-02	1.56E-02	1.7	1.75E-02
LLI wall	1.36E-02	4.0	1.28E-02	1.55E-02	0.8	1.52E-02
Small intestine	1.24E-02	3.2	1.22E-02	1.37E-02	0.5	1.37E-02
Stomach wall	1.11E-02	2.3	1.11E-02	1.20E-02	0.4	1.20E-02
ULI wall	1.25E-02	2.4	1.27E-02	1.34E-02	0.6	1.36E-02
Heart wall	1.76E-02	6.5	1.77E-02	2.09E-02	9.2	1.26E-02
Kidneys	2.96E-02	8.4	7.07E-02	1.87E-02	9.1	4.06E-02
Liver	5.84E-02	12.6	8.40E-02	3.14E-02	6.3	4.32E-02
Lungs	1.64E-02	6.7	1.11E-02	2.17E-02	12.0	1.23E-02
Muscle	9.82E-03	3.1	9.42E-03	1.10E-02	0.4	1.08E-02
Ovaries	1.38E-02	4.0	1.30E-02	1.57E-02	0.7	1.54E-02
Pancreas	1.40E-02	0.7	1.55E-02	1.37E-02	0.7	1.43E-02
Red marrow	9.71E-03	2.0	9.41E-03	1.04E-02	0.4	1.03E-02
Osteogenic cells	1.39E-02	3.6	1.26E-02	1.57E-02	0.8	1.53E-02
Skin	7.22E-03	3.7	6.73E-03	8.17E-03	0.7	8.00E-03
Spleen	1.52E-02	7.9	1.50E-02	1.15E-02	0.5	1.16E-02
Testes	1.58E-02	6.6	1.13E-01	1.25E-02	5.1	6.41E-02
Thymus	9.26E-03	3.5	8.39E-03	1.09E-02	0.8	1.01E-02
Thyroid	8.86E-03	4.1	7.62E-03	1.01E-02	0.9	9.63E-03
Urinary bladder wall	1.54E-01	0.3	1.54E-01	1.56E-01	0.0	1.56E-01
Uterus	1.90E-02	2.9	1.82E-02	2.09E-02	0.5	2.06E-02
Total body	1.19E-02	0.0	1.21E-02	1.18E-02	0.4	1.19E-02
Effective dose (mSv/MBq)	1.88E-02	2.7	2.72E-02	1.84E-02	1.9	2.19E-02

Human absorbed dose (mGy/MBq) and effective dose for ICRP 103 weighting factors (mSv/MBq) derived by animal tissue distribution (TD) and dynamic imaging based on single sphere segmentation (both bladder fraction 0.5) for the standard 70-kg male phantom including no extrapolation (NE) estimation based on pure animal data.

In Table 3, the variation of the urinary bladder wall absorbed dose when using fractions of 0.3 to 0.5 as previously described is shown as well as its impact on the effective dose for both methods. When reducing the voiding interval to 2 h, the absorbed dose decreases to 9.21E-02 mGy/MBq (TD) for a bladder fraction of 0.5 and to 5.94E-02 mGy/MBq (TD) for a bladder fraction of 0.3.

**Table 3:** Influence of bladder fraction on urinary bladder wall dose (UBW) and effective dose (ED)

**Dose**		**TD**	**Dynamic single**
Bladder fraction 0.3	UBW	9.67E-02	9.85E-02
	ED	1.66E-02	1.63E-02
Bladder fraction 0.4	UBW	1.26E-01	1.28E-01
	ED	1.77E-02	1.74E-02
Bladder fraction 0.5	UBW	1.54E-01	1.56E-01
	ED	1.88E-02	1.84E-02

Variation of UBW absorbed dose in mGy/MBq and ED for ICRP 103 weighting factors (mSv/MBq) for different bladder fractions and a voiding interval of 4 h.

## Discussion

The tissue distribution of [^18^F]UCB-H was obtained by traditional organ harvesting at several time points in mice as well as by dynamic microPET imaging to compare the results of both methods. Dynamic microPET imaging is an interesting alternative to organ harvesting. Organ harvesting requires many animals and can be labor intensive. In dynamic microPET imaging, the complete biodistribution can be obtained within a single scan. Furthermore, it allows sampling of the activity distribution with a much higher time resolution, thus catching early blood uptake peaks in organs that are well perfused. Such peaks could substantially contribute to organ radiation. However, when comparing the residence times obtained with all three techniques (Table 1), dynamic imaging (dynamic whole) alone significantly over- or underestimated the activity within organs. This effect could be explained by known imaging limitations in very small volumes such as partial volume effect or spill over from nearby organs. Low activity organs (i.e., the heart and lung) were overestimated by 36 to 89% in the microPET images presumably due to spill over from neighboring high activity organs (i.e., the liver). All other organs were underestimated in microPET images by 43 to 50%, which could be related to partial volume effect. Deriving TACs from small sphere VOIs in the center of organs (dynamic single) should have reduced the impact of partial volume effect and slightly improved quantification. However, underestimations of 49 to 53 % and overestimations of 33 to 55% remained. A further improvement in quantification might have been achieved by applying partial volume correction based on the inversion of geometric transfer matrix [18], but it requires the point spread function of the system and accurate anatomical information (ideally from MRI). Another, simpler approach to overcome these limitations that could be useful is a combined method where animals are sacrificed after dynamic imaging, and the activity of the harvested organs is quantified with a gamma well counter to cross-calibrate organ activity measured with microPET. Kesner et al. applied this approach to dosimetry in rats [15]. An investigation of this approach in mice, where size dependent effects are more eminent than in rats, will be part of future studies. Furthermore, movement artifacts arising due to rapid respiration and heart motion could be avoided by gated scanning [19]. However, it should be noted that gating decreases signal to noise ratios and leads to longer and fewer frames. An iodine-based contrast agent was used to aid organ delineation in the microCT scans. Although of great use in organ delineation, this contrast agent is most likely cleared via the same pathways as the tracer and might thus have altered uptake in liver and kidneys. The effect of IP contrast agent on residence times in the TD method will be considered in future studies.

The correlation between the extrapolated dose estimates derived by TD and dynamic single was *r* = 0.9666 (*p* < 0.0001) and *r* = 0.9603 (*p* < 0.0001) between TD and dynamic whole. The largest absolute differences in absorbed dose were found in brain, liver, and kidneys, which were underestimated in dynamic imaging by 72% (expressed as percentage of absorbed dose derived by organ harvesting), 46% and 37%, respectively. Since the brain data in dynamic imaging were derived from the remainder, the large deviation in absorbed dose for brain is a logical consequence of the methodology, as opposed to liver and kidneys, which were clearly derived by both methods. However, the effective dose deviated by only approximately 2%.

In comparison to the effective dose for 18F-FDG using the 70-kg adult male phantom, [^18^F]UCB-H remained below the reported effective dose for FDG of 2.1E-02 to 2.9E-02 mSv/MBq [20] with values (based on ICRP 103) of 1.88E-02 mSv/MBq (TD) and 1.84E-02 mSv/MBq (dynamic single). Based on the old tissue weighting factors from ICRP 60, the effective dose was 2.18E-02 mSv/MBq (TD) and 2.15E-02 mSv/MBq (dynamic single). These values are also well below the limits given by the ICRP [21]. When assuming a smaller bladder fraction of 0.3, the effective dose based on TD is decreased to an average of 1.66E-02 mSv/MBq (dynamic single 1.63E-02 mSv/MBq, both ICRP 103) for the standard 70-kg male. Assuming a shorter voiding interval (pre-scan hydration of the subject) of 2 h, the effective dose further decreases to 1.65E-02 mSv/MBq (TD and ICRP 103; 1.61E-02 mSv/MBq for dynamic single) for a bladder fraction of 0.5 and 1.53E-02 mSv/MBq (TD and ICRP 103; 1.49E-02 mSv/MBq for dynamic single) for a bladder fraction of 0.3.

The dose limits described by the US Food and Drug Administration (expressed in equivalent dose, equal to absorbed dose in this case, radiation weighting factor equal to 1) state that 30 mSv per scan should not be exceeded, or an annual dose of 50 mSv for whole body, blood forming organs, lens of the eye and gonads. The limits for all other organs are 50 mSv (single scan) and 150 mSv (annual) [22]. Therefore, for research in the USA, the maximum single injected dose of [^18^F]UCB-H allowed (assuming a bladder fraction of 0.5 and ICRP 103 factors) is 325 MBq, while the maximum annual dose is 974 MBq, with the urinary bladder being the critical organ for both (values derived from TD; dynamic imaging yields very similar values of 321 and 962 MBq). When decreasing the bladder fraction to 0.3, the critical organ remains the urinary bladder, but the single and annual doses increase to 517 MBq for single injection and 1,551 MBq for annual injection (508 MBq/1,523 MBq dynamic imaging). European regulations propose that the effective dose should not exceed 10 mSv per scan [21]. Based on this more stringent criterion, the maximum injectable dose per scan derived by organ harvesting would be 532 MBq per subject (543 MBq dynamic single). If the bladder fraction is only 0.3, the maximum injectable dose is 601 MBq (614 MBq dynamic single). However, the presently derived injection limits represent a worst case scenario due to assumptions made, such as physical decay only after the last measured time point or the relatively high fraction of injected activity cleared via urinary pathways. First human clinical trials are indispensable for confirming injection limits.

In displacement studies, subjects are injected at least twice in a short time interval in order to detect the baseline state and the activation state, or displacement [23]. Therefore, a limit of 300 MBq per scan will keep radiation exposure below harmful limits with an annual limit of three injections for practice in the USA. However, based on the high amount of activity reaching the brain [2], the injection of 200 MBq per scan ought to be sufficient to provide diagnostically useful images with [^18^F]UCB-H.

## Conclusions

In this study, it was shown that the novel SV2A radiotracer [^18^F]UCB-H meets the standard regulations regarding radiation dose for use in human clinical trials. A maximum single injectable dose of approximately 325 MBq per scan was estimated based on the classical organ harvesting technique. Dynamic imaging results by microPET showed significant deviations from organ harvesting results for single-organ absorbed doses, indicating that accurate quantification in such small volumes as mice organs is limited. However, the effective dose derived by microPET deviated by only 2% from the classical organ harvesting result.

## Competing interests

All authors declare that they have no competing interests.

## Authors’ contributions

FB carried out the experiments, participated in the design of the study, and drafted the manuscript. GW carried out the experiments and participated in the design of the study. MAB participated in the design of the study and interpretation of data. JA carried out radiochemistry. NM, TB, FM, and AV revised the manuscript for important intellectual content. FG and CL helped carrying out radiochemistry. AL and ES revised the manuscript for important intellectual content. AS and AP participated in the design of the study, interpretation of data, and revised the manuscript for important intellectual content. All authors read and approved the final manuscript.

## Supplementary Material

Additional file 1 **TACs (dynamic whole).** Time-activity curves derived by whole organ segmentation. Click here for file

Additional file 2 **TACs (dynamic whole).** Time-activity curves derived by whole organ segmentation (zoom in). Click here for file

Additional file 3 **Mean doses (dynamic whole).** Mean absorbed doses in mGy/MBq including effective dose in mSv/MBq (ICRP 103) from whole organ segmentation. Click here for file

Additional file 4 **Effective doses (ICRP 60).** Mean effective doses from old tissue weighting factors from ICRP 60. Click here for file
