# Supplementary figures and images for: Preclinical radiation dosimetry for the novel SV2A radiotracer [18F]UCB-H
Source: EJNMMI Res. 2013 May 7;3:35. doi: 10.1186/2191-219X-3-35 (PMC3655042; doi:10.1186/2191-219X-3-35)

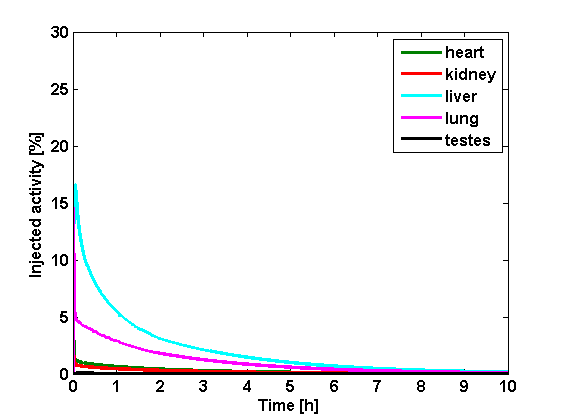

Supplement: Additional file 1 — TACs (dynamic whole). Time-activity curves derived by whole organ segmentation. [file 2191-219X-3-35-S1.tiff]

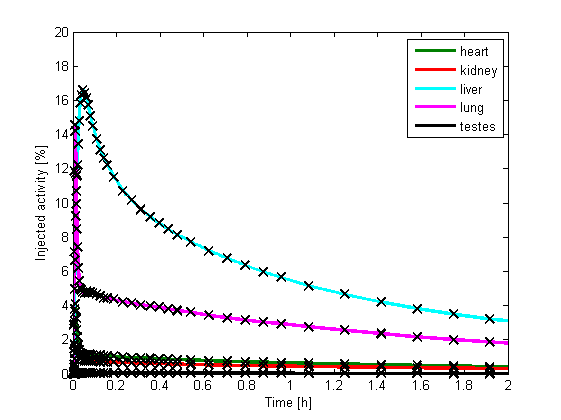

Supplement: Additional file 2 — TACs (dynamic whole). Time-activity curves derived by whole organ segmentation (zoom in). [file 2191-219X-3-35-S2.tiff]
